# Application of engineered CRISPR/Cas12a variants with altered protospacer adjacent motif specificities for the detection of isoniazid resistance mutations in *Mycobacterium tuberculosis*

**DOI:** 10.1128/spectrum.00165-25

**Published:** 2025-09-03

**Authors:** Peng Liu, Jinping Zhang, Yaqi Gong, Wenqi Liu, Guohui Xiao, Juan Liang, Xuan Wang, Jing Bi, Guoliang Zhang

**Affiliations:** 1 National Clinical Research Center for Infectious Diseases, Guangdong Provincial Clinical Research Center for Tuberculosis, Shenzhen Third People's Hospital, Southern University of Science and Technology 255310, Shenzhen, China; 2 Wuhan Pulmonary Hospital, Wuhan Tuberculosis Prevention and Control Institute, The Hubei Branch of the National Clinical Research Center for Infectious Disease, Wuhan, China; 3 Intensive Care Unit, Liyuan Hospital, Tongji Medical College, Huazhong University of Science and Technology, Wuhan, China; 4 Clinical Laboratory Experiment Center, Hangzhou Red Cross Hospital 117871, Zhejiang, China; National Institute of Immunology, New Delhi, India

**Keywords:** isoniazid, drug-resistant, tuberculosis, clustered regularly interspaced short palindromic repeats, Cas12a_RR

## Abstract

Drug-resistant tuberculosis (TB) is a major global public health concern. Although isoniazid is currently considered one of the most effective first-line drugs for TB treatment, its efficacy is limited by the emergence of resistance. Therefore, it is imperative to develop new methods for detecting drug-resistant TB. In this study, we developed a nucleic acid detection system based on the clustered regularly interspaced short palindromic repeat (CRISPR) Cas12a_RR protein. The system combines recombinase polymerase amplification with an engineered CRISPR/Cas12a_RR protein to enable rapid and specific detection of the *katG* G944C mutation in isoniazid-resistant *Mycobacterium tuberculosis* (*Mtb*). It could detect the target DNA at concentrations as low as 1% in a mixed sample. Compared with TaqMan quantitative polymerase chain reaction and DNA sequencing, the CRISPR/Cas12a_RR system demonstrated superior detection performance in terms of sensitivity, specificity, and cost-effectiveness. Furthermore, it effectively differentiated between drug-resistant *Mtb* strains from wild-type *Mtb* strains in clinically isolated samples, with the entire detection process completed in 60 min. In conclusion, the CRISPR/Cas12a_RR detection system offers a novel, rapid, simple, sensitive, and specific approach for identifying isoniazid-resistant *Mtb*, with significant potential for clinical application, particularly in resource-limited settings.

**IMPORTANCE:**

This study presents a novel method for detecting isoniazid-resistant *Mycobacterium tuberculosis* (*Mtb*) using clustered regularly interspaced short palindromic repeat (CRISPR)/Cas12a mutants, offering rapid detection, cost-effectiveness, and high specificity, and thereby providing a promising new avenue for detecting isoniazid-resistant *Mtb*.

## INTRODUCTION

Tuberculosis (TB) is a chronic infectious disease caused by *Mycobacterium tuberculosis* (*Mtb*) (1–3). According to the 2024 World Health Organization Global TB Report, there were an estimated 10.80 million new TB cases globally in 2023, resulting in 1.25 million deaths (1). Currently, Bacillus Calmette-Guérin is the only vaccine against TB infection, although it is only effective in children and has a protection period of 10–20 years (1, 4–6). Antibiotics play an indispensable role in TB prevention and control. However, extensive and irrational use of antibiotics has led to the emergence of drug resistance, especially against isoniazid and rifampin, the commonly used first-line anti-TB drugs. Mutations in the *katG* gene, including G944C (S315T) and G944A (S315N), are primarily responsible for isoniazid resistance. The G944C (S315T) mutation in the *katG* gene accounts for 50%–90% of all cases of isoniazid resistance (7, 8). In 2023, the estimated number of multidrug-resistant (MDR-TB) or rifampicin-resistant (RR-TB) TB cases was 400,000 (1). Therefore, the emergence of drug-resistant TB poses a significant challenge to public health systems, contributing to increasing mortality rates and hampering disease prevention and control (1, 9). Heterogeneous drug resistance in *Mtb* refers to the presence of bacteria that are both susceptible and resistant to anti-TB drugs. This phenomenon is considered the initial step toward the development of full drug resistance (10–12).

The current bacteriological tests available for detecting drug-resistant TB include *Mtb* phenotypic drug susceptibility testing (pDST), real-time quantitative polymerase chain reaction (qPCR), GeneXpert MTB/RIF, whole-genome sequencing (WGS), next-generation sequencing (NGS), and Sanger sequencing (1, 13–18). Among these, conventional mycobacterial culture and pDST remain the gold standards for diagnosis in clinical practice (1). However, these methods are time-consuming, requiring weeks to obtain accurate results and demanding specialized equipment and technical expertise, leading to potential delays in treatment. Molecular testing has emerged as a promising approach to bridge the diagnostic gap. Compared with pDST, it provides significantly faster results (19, 20). However, the implementation of WGS and NGS is hindered by relatively high equipment costs, which complicates their deployment in resource-limited settings. Currently, the most commonly employed NGS strategy for the detection of drug-resistant TB in clinical practice is targeted NGS (tNGS). tNGS integrates the amplification of selected genes with NGS, enabling the detection of only pre-defined targets (19, 20). Nevertheless, owing to the low positive detection rate of drug-resistant TB and the lack of rapid and sensitive detection methods, a large number of patients with drug-resistant TB do not receive timely formal treatment, resulting in disease transmission and death. Therefore, there is an urgent need for a rapid and innovative method for detecting drug-resistant *Mtb*.

Clustered regularly interspaced short palindromic repeat (CRISPR), an immune defense system used by prokaryotes to resist the invasion of foreign genetic material, has been engineered as an efficient gene-editing tool (21, 22). Recent research has shown that certain class II Cas proteins, such as CRISPR/Cas13a and CRISPR/Cas12a, possess “incidental cleavage” activity, enabling the nucleic acid detection of several important pathogens such as Zika virus, dengue virus, and TB (23–26). Nucleic acid detection technology based on the CRISPR/Cas system offers advantages such as high sensitivity, specificity, efficiency, and low cost. Cas proteins, guided by the CRISPR RNA (crRNA), effectively identify and cleave target DNA sequences (27), thereby enhancing detection accuracy. Additionally, CRISPR technology demonstrates an impressive level of sensitivity, with studies indicating that it can detect the Zika virus and dengue virus in patient samples at concentrations as low as 1 copy per microliter (25). This capability further facilitates early disease diagnosis and the monitoring of transmission. Moreover, the simplicity of operation, the lack of the need for large instruments, and rapid performance make CRISPR technology a valuable tool. CRISPR/Cas12a is the most commonly used effector protein in nucleic acid detection methods. However, it has sequence specificity for substrate recognition because it can only identify motifs containing TTTV (27–29), limiting the broader implementation of this method. Recent studies have shown that mutating two key amino acids at positions 532 and 595 of the CRISPR/Cas12a protein to G532R and K595R, respectively, results in a variant referred to as CRISPR/Cas12a_RR, which can recognize unconventional protospacer adjacent motif (PAM) sequences, such as TTCC, CTCC, and TCCC in target sequences. Recombinant polymerase amplification (RPA) offers several advantages over PCR, such as the need for simple equipment, requiring only a thermostat or water bath, and ease of use by eliminating complex temperature adjustments. The results are obtained within 20 min, enabling rapid screening and diagnosis. Furthermore, isothermal amplification is resilient to temperature fluctuations and suitable for wider temperature ranges and resource-limited environments, as well as direct detection of amplification products, further enhancing experiment efficiency (30, 31).

Based on the engineered CRISPR/Cas12a protein (28, 32), in this study, we used a combination of single-enzyme digestion and RPA to develop a novel protein detection system using the CRISPR/Cas12a_RR mutation, which could identify unconventional target sequences of PAMs. This method was first used to detect the *katG* G944C mutation in isoniazid-resistant *Mtb* with high specificity. Given the overall efficiency of this detection system, it is likely to accelerate the timely diagnosis and treatment of patients with drug-resistant TB. This study presents a precise, rapid, and straightforward testing method for frontline healthcare workers, particularly in economically underdeveloped regions.

## MATERIALS AND METHODS

### Clinical samples and ethics statement

This study was approved by the Ethics Committee of the Shenzhen Third People’s Hospital (approval no. 2023-011-02). Clinical isolates of *Mtb* were collected from untreated patients with active TB. Prior to selection, Sanger sequencing confirmed the presence of the G944C mutation in the *katG* gene of isoniazid-resistant *Mtb*. All clinical samples were collected and processed in strict accordance with the procedures recommended by the World Health Organization and the Third People’s Hospital of Shenzhen. The samples were inoculated on 7H10 agar plates supplemented with 10% oleic acid-albumin-dextrose-catalase and cultured for 2 weeks (33, 34). The *Mtb* colonies were suspended in phosphate-buffered saline and subsequently heated in a water bath at 100°C for 10 min to heat-inactivate them in a Biosafety Level 3 laboratory. Subsequently, DNA was extracted using the Tuberculosis DNA extraction kit (Gene Optimal, China).

### dsDNA preparation

The *katG* G944C (S315T) mutation of INH-resistant *Mtb* was designed according to the sequence of the H37Rv standard strain in Mycobrowser (https://mycobrowser.epfl.ch/genes/Rv1908c). The mutant and wild-type fragments were synthesized by Sangon Biotech (Sangon, China), purified, recovered after PCR amplification, and stored at –20°C until use. The PCR product was purified on a silica gel column (Omega Bio-Tek, USA). First, the molecular weight of the *katG* gene fragment was calculated, and *katG* template samples containing 1 × 10^11^, 1 × 10^10^, 1 × 10^9^, 1 × 10^8^, and 1 × 10^7^ copies/μL were obtained via gradient dilution. We then mixed the wild-type *katG* fragment with the mutant gene fragment in varying ratios to generate test templates with mutation rates of 100%, 10%, 1%, 0.1%, 0.01%, and 0%. The DNA template was first amplified by RPA and incubated at 37°C for 20 min. After purification, it was stored at –20°C for future use.

### crRNA design and screening

Based on the *katG* wild-type and G944C mutant genes of *Mtb*, several specific crRNAs positioned adjacent to the PAM sequences were designed, featuring 23-base pair (bp) core sequences that encompass the mutation sites. By introducing a base mismatch, a bubble of bp was formed in the absence of the target bp, enabling a more accurate detection at the desired site. SnapGene 3.2.1 and Primer Premier 5 were employed for designing the crRNA, primer, and probe sequences. crRNAs were synthesized by Sangon Biotech. The names and core sequences of various crRNAs are detailed in Table S1.

### Design and screening of RPA primers for the *katG* gene

RPA primers were designed to amplify the products containing target DNA sequences in accordance with the requirements of RPA. RPA amplification primers with lengths of 30–37 bp were designed and synthesized by Sangon Biotech, and the different primer names and sequences used are listed in Table S2. The amplified *Mtb katG* fragment was added to the CRISPR/Cas12a_RR reaction system and incubated at 37°C for 15 min. Fluorescence was detected using a microplate reader and a 485-nm blue light reader, and the primer with the strongest fluorescence signal was selected for subsequent experiments.

### Isothermal amplification

The *katG* gene was subjected to RPA using a commercial ERA kit (GenDx, China) according to the manufacturer’s instructions. Briefly, a 50-µL reaction system containing 20 µL solubilizer, 2 µL DNA, 2.5 µL forward primer (10 µM), 2.5 µL reverse primer (10 µM), 21 µL ddH_2_O, and 2 µL activator was incubated at 37°C for 20 min. The amplified products were purified, recovered, and stored at –20°C until use.

### Digestion reaction

The AciI enzyme cleavage site in the *Mtb katG* wild-type gene at position 944 is affected by a mutation from G to C. This mutation results in the loss of the AciI cutting site, which can be used to selectively cut the *katG* wild gene. Consequently, this alteration decreased the presence of the wild-type gene template and enhanced the presence of the mutant gene template, thereby aiding in the amplification of *katG* G944C mutant genes during RPA. The DNA template was digested with AciI restriction endonuclease, followed by amplification using RPA. The reaction was incubated at 37°C for 20 min, and the amplified DNA was subsequently purified, recovered, and stored at –20°C for future use.

### Deep sequencing

RPA products were sent to Sangon Biotech for sequencing and library preparation. Briefly, amplification of the target sequence was performed using a two-step PCR method compatible with Illumina sequencing library preparation. The PCR products were purified and recovered using AMPure XP magnetic beads. Paired-end sequencing of the library was performed using the NovaSeq6000/MiSeq PE150/PE300 system (Illumina, USA).

### TaqMan qPCR

TaqMan qPCR primers and probes were designed and synthesized by Sangon Biotech. The probes were complementary to the *katG* G944C template with the 5′ reporter dye FAM and 3′-MGB. The 20-µL TaqMan qPCR system included a 10-µL 2× Taq Pro HS Universal Probe Master Mix (Vazyme, China), 0.2-µL TaqMan probe (10 µM), 0.4-µL qPCR forward primer (10 µM), 0.4-µL qPCR reverse primer (10 µM), 1-µL template DNA, and 8-µL ddH_2_O. PCR cycling conditions were 37°C for 2 min, 95°C for 30 s, and 45 cycles of 95°C for 10 s, and 60°C for 30 s. Sequences of the qPCR probes and primers are listed in Table S3.

### CRISPR/Cas12a detection reaction

**Fig 1:**
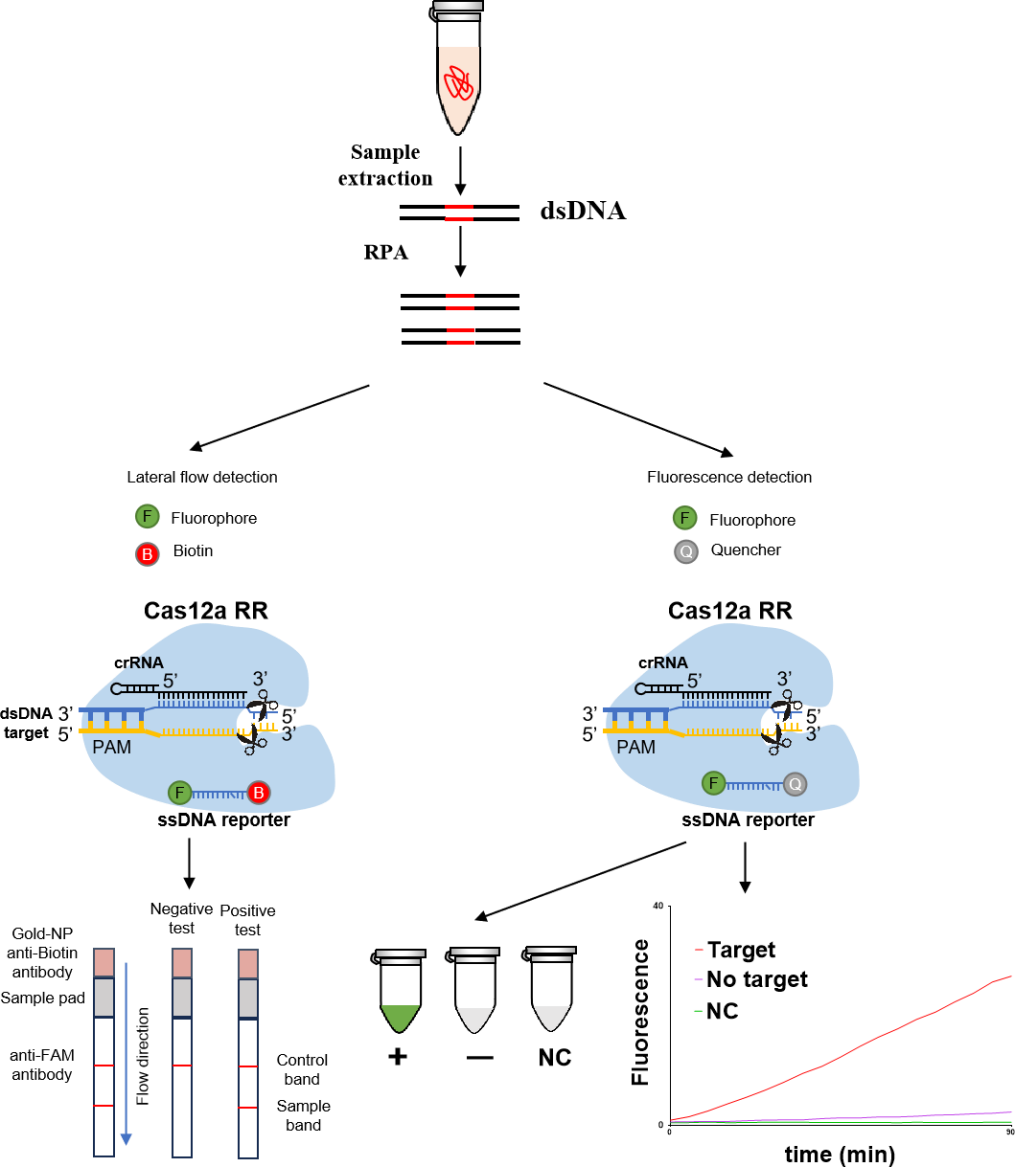
CRISPR/Cas12a_RR system for detecting INH-resistant *Mtb*. Schematic representation of the CRISPR/Cas12a_RR system for detecting isoniazid-resistant *Mtb*. We designed specific crRNAs targeting the mutation site of *katG* in isoniazid-resistant *Mtb*. When CRISPR/Cas12a_RR proteins cleave the double-stranded DNA guided by specific crRNAs, they induce robust nonspecific trans-cleavage of single-stranded DNA (ssDNA). The ssDNA (sequence TTATT) reporter emits green fluorescence when the ssDNA containing the quenched fluorophore is cleaved. The CRISPR/Cas12a RR immunochromatographic detection system employs a FAM-biotin reporter gene. In negative samples, gold nanoparticles (NPs) attach to the uncleaved reporter gene and are retained at the control line by anti-FAM antibodies. Conversely, in positive samples, the reporter gene is cleaved, enabling NPs to bind to the detection line through biotin, which leads to a diminished signal at the control line.

Detection based on CRISPR/Cas12a was conducted in accordance with previous descriptions, with modifications (32, 35). The CRISPR/Cas12a protein used in the detection system for isoniazid resistance in *Mtb* was produced using an *Escherichia coli* expression system. The genes encoding CRISPR/Cas12a were optimized for codons and inserted into the pET-28a expression vector. The soluble form of CRISPR/Cas12a, which includes a TEV cleavage site and a 6× His-tag at the C-terminus, was expressed and purified following previously described protocols (18) with subsequent modifications. Protein expression was conducted using the *E. coli* BL21 (DE3) strain, with induction at 1 mM IPTG for 16 h at 16°C. The bacterial cells were then collected and lysed using a high-pressure cell disruptor in a lysis buffer containing (500 mM NaCl, 25 mM Tris-HCl, pH 8.0, 10% [vol/vol] glycerol, and 0.5 mM PMSF). The soluble CRISPR/Cas12a was isolated using an Ni-NTA resin and treated with TEV protease at 4°C overnight to eliminate the C-terminal His-tag. The proteins were further purified using the Superdex 200 Increase filtration column (GE Healthcare Life Sciences, USA) in conjunction with fast protein liquid chromatography. The purified CRISPR/Cas12a protein was concentrated into a storage buffer consisting of (500 mM NaCl, 50 mM Tris-HCl, pH 7.5, 2 mM DTT, and 10% [vol/vol] glycerol), quantified using the BCA Protein Assay Kit (Beyotime, China), and subsequently stored at −80°C until required. The CRISPR/Cas12a_RR mutant protein was created by mutating two crucial amino acids at positions 532 and 595 of the CRISPR/Cas12a protein to G532R and K595R, respectively. Reactions were performed in a 20 µL solution containing 200 ng CRISPR/Cas12a_RR protein, 1 µM crRNA, 2 µL sample DNA, 25 pM single-stranded DNA (ssDNA) FQ probe sensor, 2 µL NEBuffer r3.1 reaction buffer (100 mM NaCl, 50 mM Tris-HCl, 10 mM MgCl_2_, and 100 µg/mL BSA [pH 7.9]) under incubation at 37°C for 15 min. The fluorescence intensity was measured using the Varioskan LUX multimode microplate reader (Thermo Fisher, USA). The fluorescence activity was monitored using a monochromator with excitation and emission wavelengths of 485 and 520 nm, respectively. A CRISPR lateral flow test strip and 485-nm blue light were used simultaneously for visual detection. Upon specific recognition of the target nucleic acid fragment by the crRNA, the reaction product solution exhibited a noticeable change in color from colorless to fluorescent green. By contrast, in the absence of target nucleic acids complementary to the crRNA, the reaction product solution retained its original colorless appearance (Fig. 1).

### CRISPR lateral flow test strip

The CRISPR/Cas12a_RR immunochromatographic assay system utilizes a FAM-biotin reporter gene for detection. In negative samples, gold nanoparticle anti-biotin antibodies bound to high concentrations of FAM-biotin reporter genes, with the conjugate blocked by anti-FAM antibodies in the control strip. Conversely, in positive samples, the FAM-biotin reporter genes were cleaved, leading to the accumulation of gold nanoparticle anti-biotin antibodies with biotin conjugates in the detection strip while being reduced in the control strip. A schematic illustration of the immunochromatographic detection process is shown in Fig. 1.

### Statistical analysis

The *t*-test was used to evaluate significant variances between the two groups. The results presented in Fig. 3 to 5 and 8 were analyzed using the one-way analysis of variance, followed by pairwise comparisons among varying conditions. The data illustrated in Fig. 6A and 7B were subjected to the two-way analysis of variance. A *P* value of <0.05 was considered statistically significant. *P* values were classified in this manner: those below 0.0001, 0.001, 0.01, and 0.05 were indicated by ****, ***, **, and *, respectively. The bar graphs indicate the means ± standard deviation. Statistical evaluations were performed using GraphPad Prism version 9.0 software.

## RESULTS

### Engineered CRISPR/Cas12a_RR protein recognizes a variety of non-traditional PAM sequences

**Fig 2:**
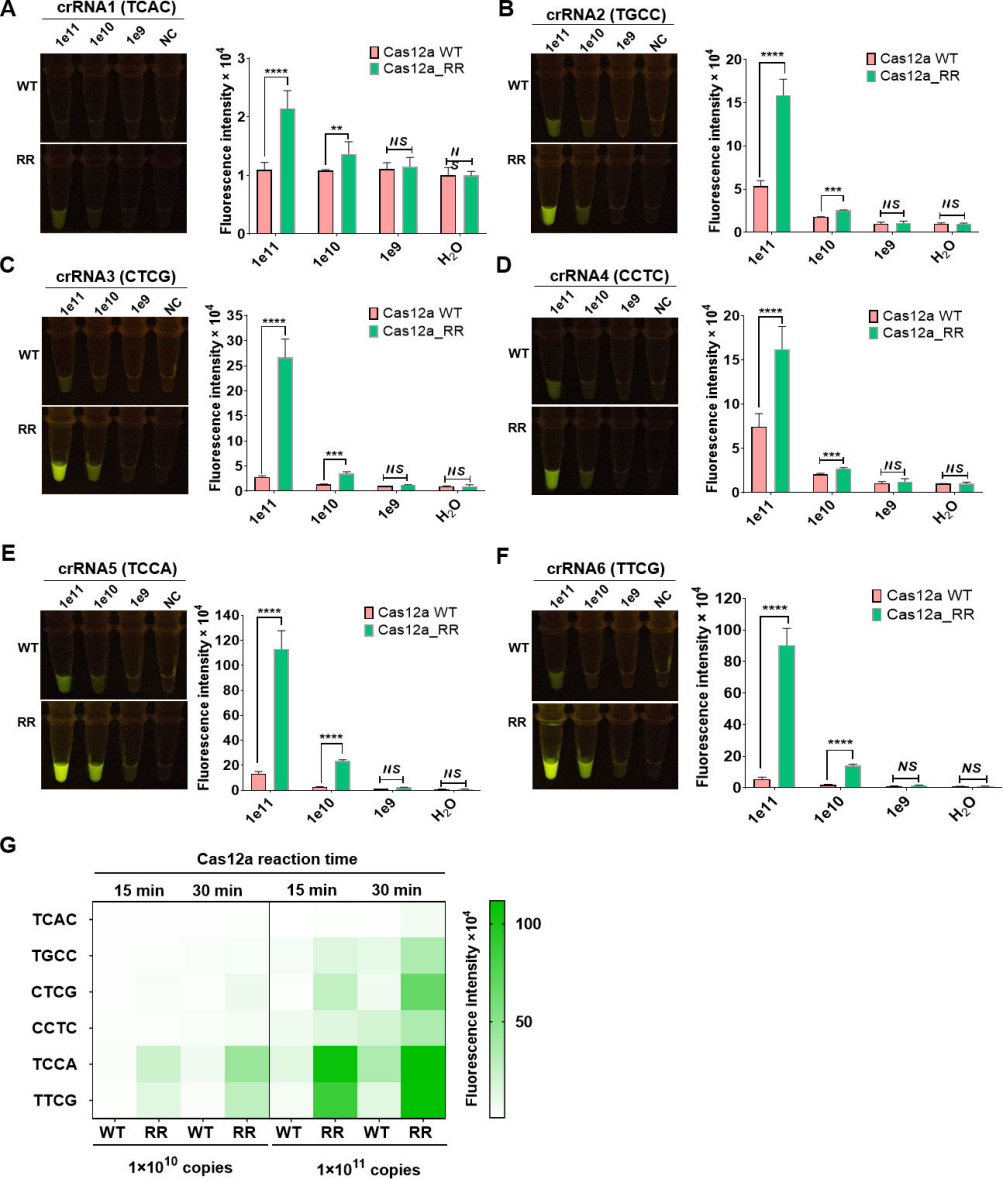
The CRISPR/Cas12a_RR protein recognizes a variety of PAMsequences. CRISPR/Cas12a RR proteins recognize the TCAC (**A**) TGCC (**B**), CTCG (**C**),CCTC (**D**), TCCA (**E**), and TTCG (**F**) targeting sequences. The fluorescence image and intensity after 15 min of reaction are shown. Fluorescence heatmap (**G**) is generated to compare the activity of CRISPR/Cas12a and CRISPR/Cas12a_RR reactions induced by different G944C-crRNAs, detecting 1 × 10^11^ and 1 × 10^10^ copies of PCR fragments. CRISPR/Cas12a reactions are monitored for 10 and 30 min. Additionally, the target sequences recognized by CRISPR/Cas12a_RR protein are identified as TCAC (**A**), TGCC (**B**), CTCG (C), CCTC (D), TCCA (**E**), and TTCG (**F**). Data arepresentedasmeans ± SD from at least three independent experiments. *P < 0.05, **P < 0.01, ***P < 0.001, and ****P < 0.0001. SD, standard deviation; PCR, polymerasechain reaction; RPA, recombinant polymerase amplification; CRISPR, clustered regularly interspaced short palindromic repeat; PAM, protospacer adjacent motif; crRNA, CRISPR RNA.

To assess the capability of the CRISPR/Cas12a_RR protein to recognize non-traditional PAM sequences, we investigated six sequences—TCAC (Fig. 2), TGCC (Fig. 2B), CTCG (Fig. 2C), CCTC (Fig. 2D), TCCA (Fig. 2E), and TTCG (Fig. 2F)—located near the G944C resistance mutation site of the *katG* gene. Corresponding crRNAs were designed for each sequence. The results demonstrated that the fluorescence intensity generated by the CRISPR/Cas12a_RR system for recognizing the target gene was significantly greater than that generated by the CRISPR/Cas12a system (Fig. 2A through G). Because the six non-traditional PAM sequences are situated near the G944C resistance mutation site of the *katG* gene, the corresponding crRNAs effectively encompass the mutation site, thus rendering these six crRNAs suitable for detecting this resistance mutation.

### Screening and optimization of crRNA at the *katG* G944C mutation site

**Fig 3:**
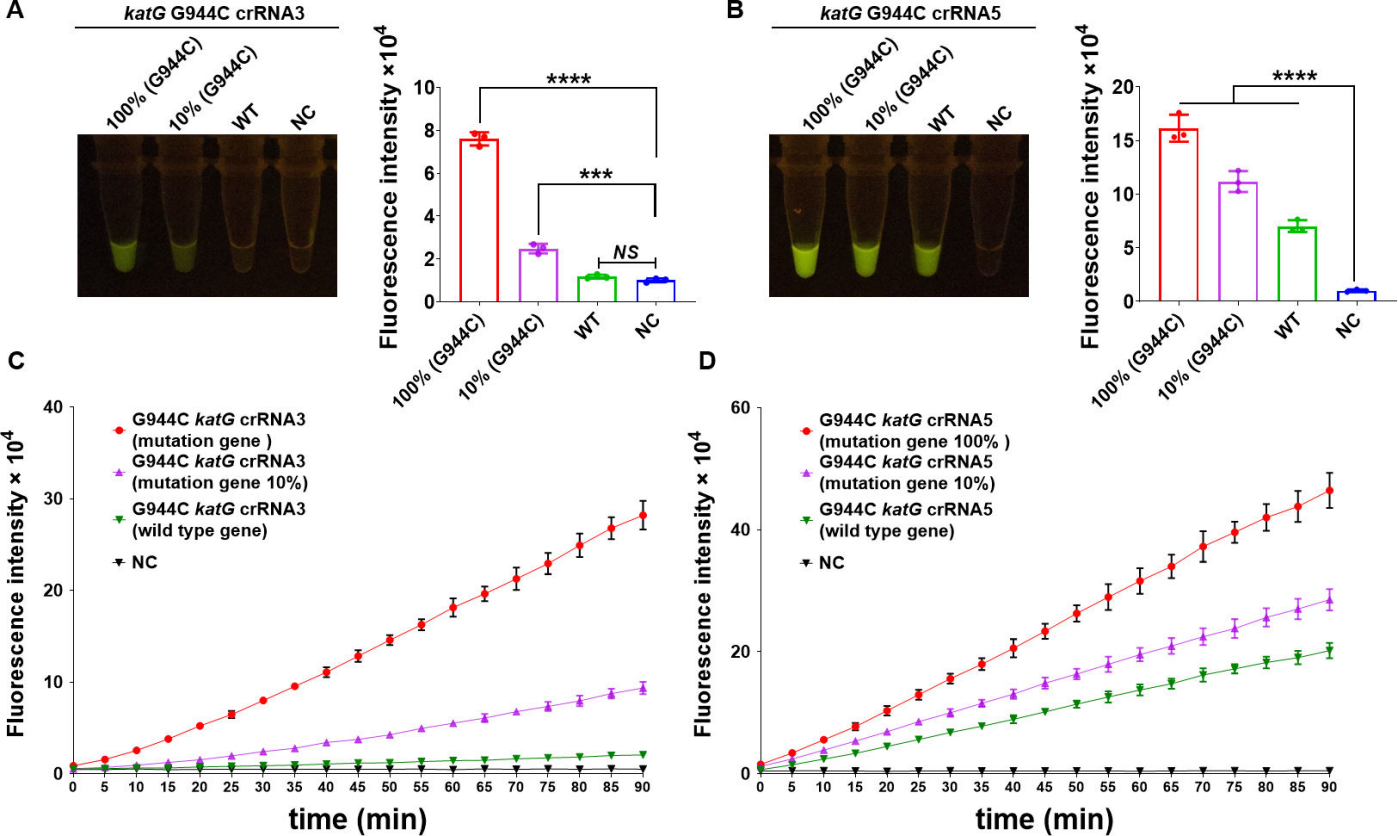
Specific crRNAs could identify the *katG* G944C gene in isoniazid-resistant *Mtb*. We assessed the specificity of crRNA in the CRISPR/Cas12a_RR system for the *katG* G944C mutant gene in INH-resistant *Mtb*. (**A and B**) Fluorescence images and intensities after 15 min of reaction. (**C and D**) Fluorescence intensities after 90 min of reaction. The concentration of the target gene used in the detection reaction is 1 × 10^11^ copies/µL. Data are presented as means ± SD from at least three independent experiments. *P < 0.05, **P < 0.01, ***P < 0.001, ****P < 0.0001.

Among the crRNAs depicted in Fig. 2, we randomly selected crRNA3 and crRNA5 for validation. To identify the crRNAs that specifically detect the *katG* G944C (S315T) mutation site, we mixed *katG* wild-type and G944C (S315T) mutant gene fragments in different proportions to obtain samples with mutation rates of 100%, 10%, and 0%. The efficiency of these crRNAs was assessed using the CRISPR/Cas12a_RR detection system, which was evaluated using a 485-nm blue light lamp and a microplate reader. The results indicated that, after a 15-min reaction, crRNA3 effectively distinguished between the wild-type and mutated genes, showing no fluorescent interference signal when identifying the wild-type *Mtb katG* gene (Fig. 3A). By contrast, crRNA5 exhibited interference signals when wild-type Mtb *katG* was detected (Fig. 3B). To further validate the specificity of the crRNAs, we dynamically monitored the entire detection reaction and found that crRNA3 maintained high specificity throughout the entire 90-min period compared with crRNA5 (Fig. 3C and D). Therefore, crRNA3 was used in subsequent experiments to identify the mutated gene fragment of *Mtb katG* G944C.

### *katG* gene fragment of *Mtb* amplified by RPA

**Fig 4:**
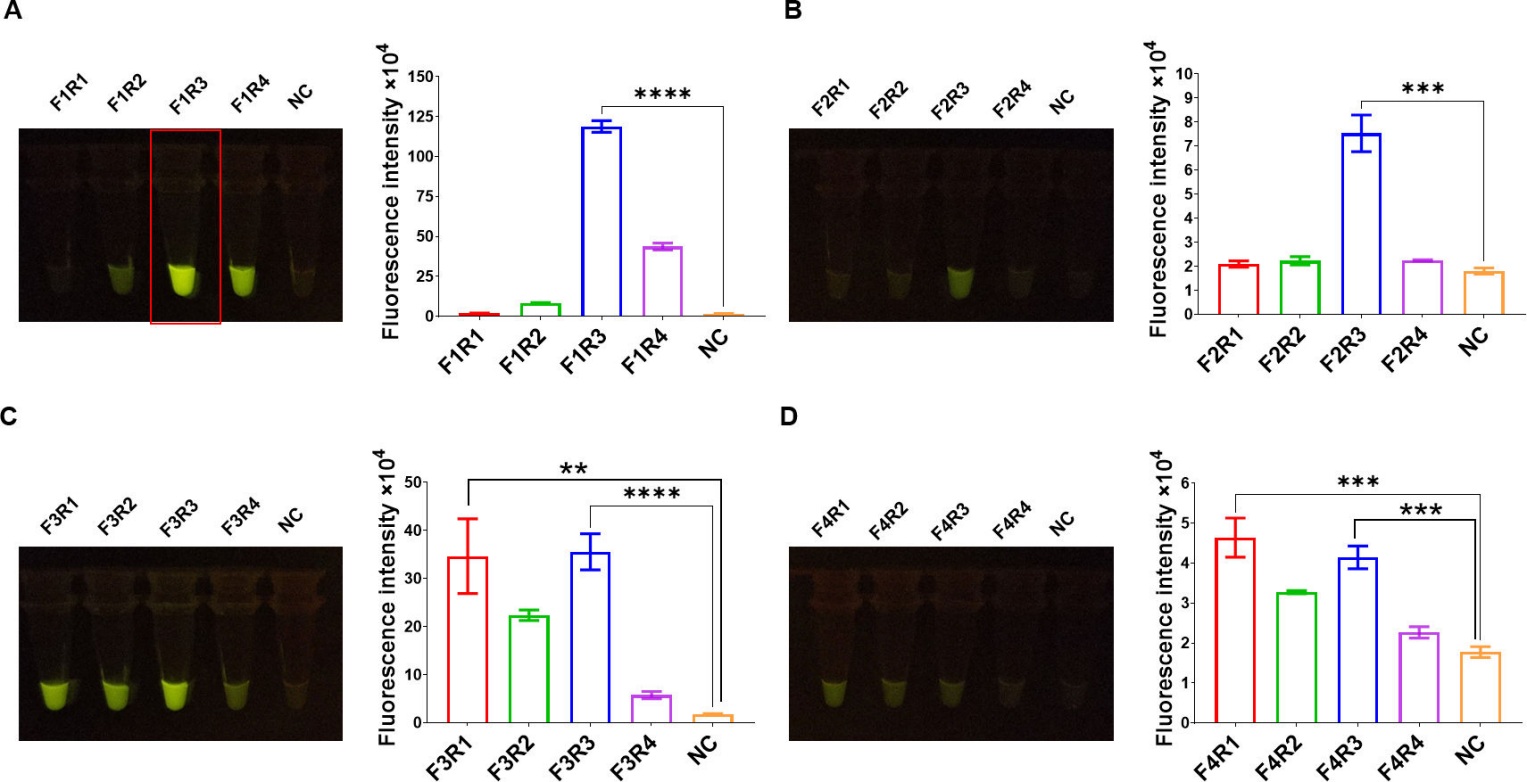
RPA can efficiently amplify the *katG* gene of *Mtb*. RPA primers are used to amplify the *katG* fragment of INH-resistant *Mtb*. The amplification efficiency of the 16 pairs of RPA primers is evaluated using the CRISPR/Cas12a_RR detection system. (**A–D**) Fluorescence images and intensities after 15 min of reaction. *P < 0.05, **P < 0.01, ***P < 0.001, ****P < 0.0001.

**Fig 5:**
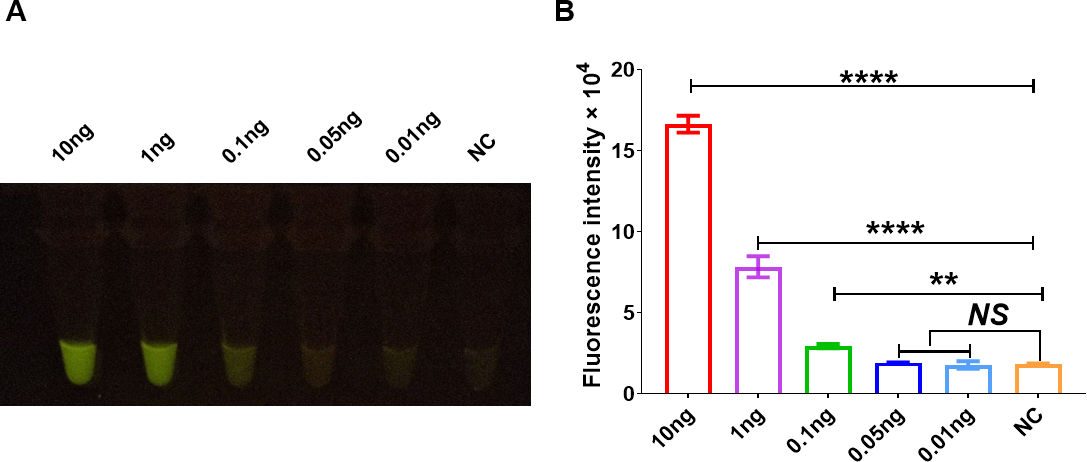
RPA technology has high sensitivity in amplifying the *katG* gene of *Mtb*. A total of 100 ng of *Mtb* genomic DNA is used in the detection reaction. The *katG* gene fragment is diluted at a gradient of 10 to 0.01 ng, and 1 µL of the sample was added to the 50-µL RPA reaction system for amplification. The sensitivity of the RPA reaction is measured using the CRISPR/Cas12a_RR fluorescence assay, which produced the fluorescence image (**A**) and intensity (**B**) after 15 min of reaction. Data are presented as means ± SD from at least three independent experiments. The DNA templates used in the amplification reactions are derived from the *Mtb* strains. *P < 0.05, **P < 0.01, ***P < 0.001, ****P < 0.0001.

In accordance with the requirements of RPA, the amplification products were designed to contain *katG* crRNA, and the lengths of RPA amplification primers were 30–37 bp. Among the 16 pairs of RPA amplification primers, the fluorescence intensity induced by the amplification products of F1R3 primers was significantly higher than that of the other primers, indicating a pronounced amplification effect (Fig. 4A through D). Therefore, this primer pair was selected for subsequent experiments. To assess the amplification sensitivity of RPA, we amplified the target gene *katG* using whole-genome templates of *Mtb* at varying concentrations. The results demonstrated that the *katG* gene fragment could be amplified from as low as 0.1 ng of the entire genome of *Mtb*, exhibiting high amplification sensitivity (Fig. 5A and B).

### CRISPR/Cas12a_RR method has high specificity in detecting the *katG* G944C mutation

**Fig 6:**
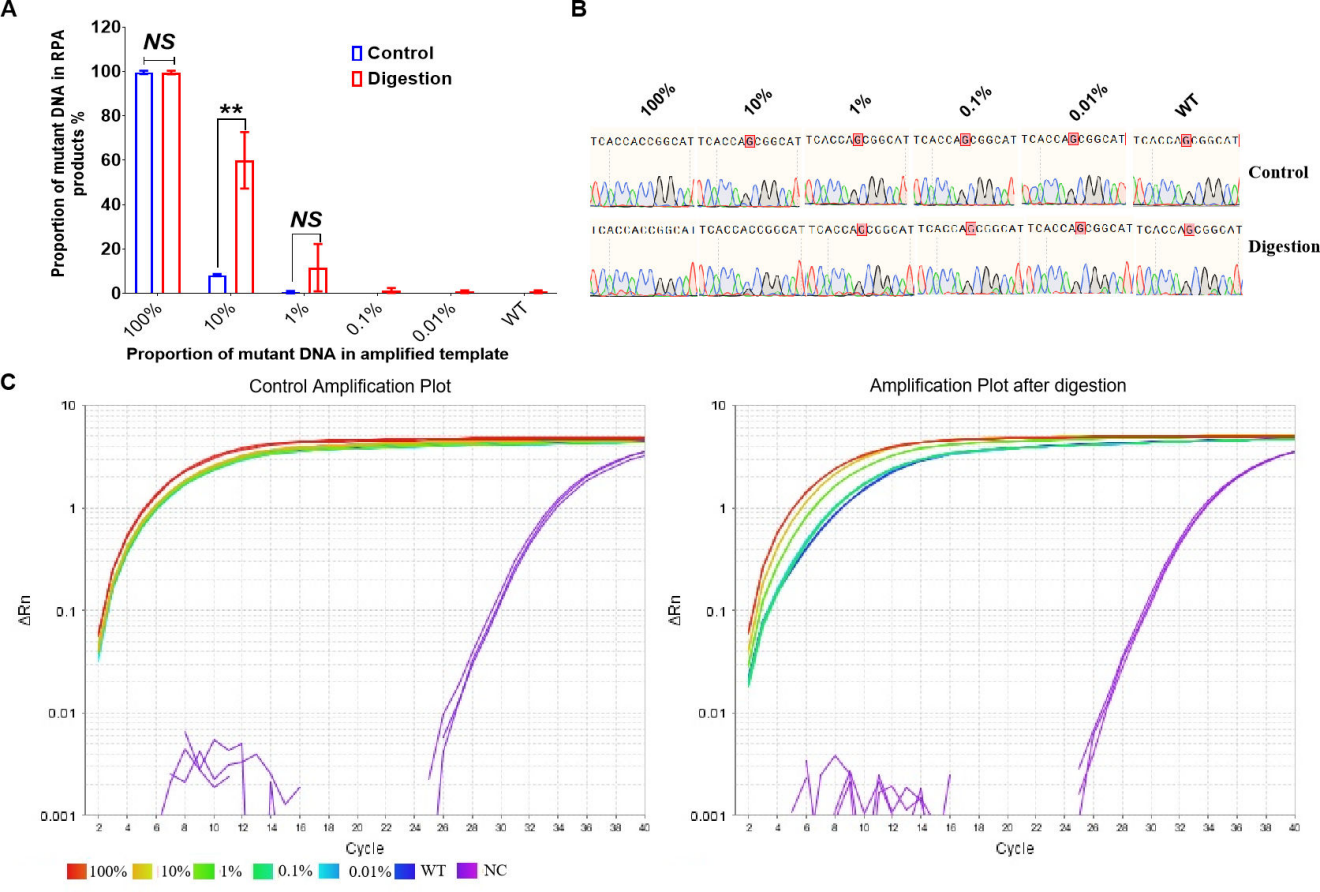
Increase in the number of mutated genes in the amplified product after digestion of the DNA template. The prepared template is digested with AciI endonuclease, followed by an RPA amplification reaction, and the RPA products were purified and subjected to DNA deep sequencing (**A**). DNA sequencing (**B**) and TaqMan qPCR (**C**) were used to detect the *katG* G944C mutation gene of isoniazid-resistant *Mycobacterium tuberculosis*. Data are presented as means ± SD from at least three independent experiments. *P < 0.05, **P < 0.01, ***P < 0.001, ****P < 0.0001.

**Fig 7:**
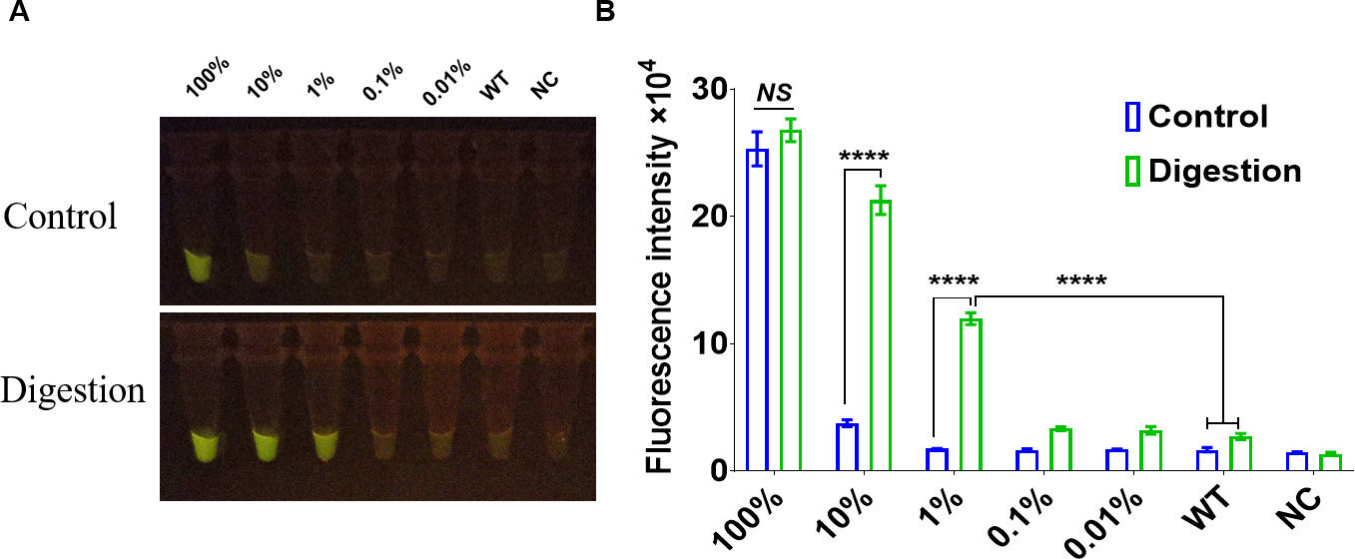
Illustration of the high specificity of the CRISPR/Cas12a_RR detection system. The specificity of the CRISPR/Cas12a_RR fluorescence assay for the *katG* gene mutation is assessed via fluorescence imaging (**A**) and intensity analysis (**B**) after 15 min of reaction. Data are presented as means ± SD from at least three independent experiments. *P < 0.05, **P < 0.01, ***P < 0.001, ****P < 0.0001.

To determine the specificity of the CRISPR/Cas12a_RR fluorescence assay for distinguishing between *katG* wild-type and *katG* G944C (S315T) mutant genes, the molecular weights of the fragments were calculated and the test template samples were prepared to contain 1 × 10^11^, 1 × 10^10^, 1 × 10^9^, 1 × 10^8^, and 1 × 10^7^ copies/μL via gradient dilution. We then mixed wild-type *Mtb* fragments with mutant fragments at varying ratios to generate assay templates with mutation rates of 100%, 10%, 1%, 0.1%, 0.01%, and 0%. We cleaved the *katG* wild-type gene using the AciI restriction site under incubation at 37°C for 20 min by restriction endonuclease treatment (Fig. S1). The DNA deep sequencing results indicated that, following the amplification of a 10% mutant DNA template following enzymatic digestion, the average content of the nucleic acid of the *katG* mutant gene increased from 8.19% to 59.94%, confirming significant enrichment (Fig. 6A). Following enzymatic digestion of wild *katG* gene fragments, DNA Sanger sequencing was used to identify the *katG* G944C drug-resistant mutation at a frequency of 10%, G944C (Fig. 6B) and TaqMan qPCR (Fig. 6C). Subsequently, 1 µL of both undigested and digested DNA templates was added to the 50 µL RPA amplification system, with ddH_2_O serving as the negative control. The system was then incubated at 37°C for 20 min, after which the amplified products were purified and recovered. Next, 10 ng of DNA product was added to the 20 µL CRISPR/Cas12a_RR detection system; after a 15-min incubation at 37°C, fluorescence intensity was assessed using a microplate reader and 485-nm blue light. The results indicated that the *katG* G944C mutant fragment could be effectively detected at a concentration of 1% using the CRISPR/Cas12a_RR system (Fig. 7A and B). These findings confirm the high sensitivity and specificity of the CRISPR/Cas12a_RR system in detecting *katG* G944C mutations in isoniazid-resistant *Mtb* strains.

### CRISPR/Cas12a_RR system rapidly detects the *katG* G944C mutation in isoniazid-resistant *Mtb* strains

**Fig 8:**
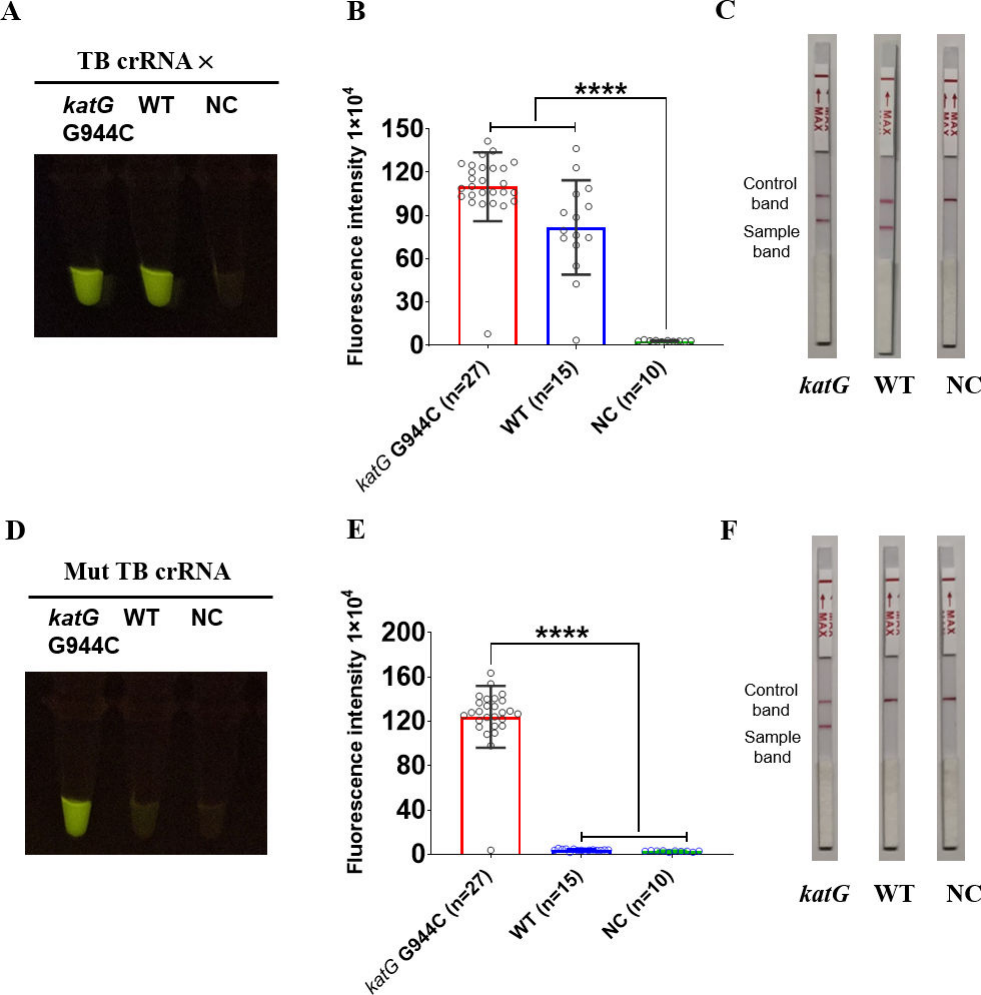
CRISPR/Cas12a_RR system can effectively distinguish isoniazid-resistant *Mtb* from wild-type *Mtb*. Fluorescence images, fluorescence intensity (**A, B, D, E**), and CRISPR lateral flow test strip (**C, F**) results after 15 min of reaction. A total of 1 ng of the *Mtb* genomic DNA template is used in the above detection reaction. Data are presented as means ± SD from at least three independent experiments. *P < 0.05, **P < 0.01, ***P < 0.001, ****P < 0.0001.

Finally, to assess the clinical applicability of the CRISPR/Cas12a_RR system for detecting the *katG* G944C mutant strain of isoniazid-resistant *Mtb*, 42 clinical isolates were collected—27 with the *katG* G944C mutation and 15 without. After 2 weeks of culture, DNA was extracted from *Mtb,* and 1 ng of the whole genomic DNA was added to the 50 µL RPA reaction system, mixed evenly, and incubated at 37°C for 20 min. We added 1 µL of RPA product to the 20 µL CRISPR/Cas12a_RR fluorescence detection system, which was mixed and incubated at 37°C for 15 min before detection using fluorometry and CRISPR lateral flow test strip assays. The results showed that both the fluorescence (Fig. 8A, B, D and E) and CRISPR lateral flow test strip methods (Fig. 8C and F) could detect and distinguish between the *katG* G944C-mutant and wild-type *Mtb*. Both the CRISPR fluorescence method and the CRISPR lateral flow test strip method can be utilized to detect and differentiate the *katG* G944C mutant strain from the wild-type *Mtb*, depending on experimental requirements. When conditions allow, the combined application of both methods can further enhance detection accuracy.

## DISCUSSION

According to a dynamic Markov model analysis, if the detection rate of drug-resistant TB in China increases from 22.5% to 50%–70% (i.e., approximately 5% per year), the prevalence of drug-resistant TB could decrease by 25.9%–36.2% by 2050 (36, 37). Therefore, there is an urgent need to develop rapid and sensitive methods for detecting drug-resistant *Mtb*.

In this study, we developed a novel CRISPR/Cas12a_RR detection system by integrating an engineered CRISPR/Cas12a_RR protein with single-enzyme digestion and RPA. The CRISPR/Cas12a protein is a crRNA-guided nuclease that specifically recognizes and cleaves nucleic acids. When the CRISPR/Cas12a protein recognizes the targeted double-stranded DNA in a sequence-specific manner, it induces strong nonspecific ssDNA trans-cleavage of fluorescent probes (29, 38–42). However, the protein can only recognize conventional thymidine nucleotide-rich PAMs (27), which are absent near the mutation sites in isoniazid-resistant *Mtb* strains. By introducing mutations in two key amino acids at positions 532 and 595 of the CRISPR/Cas12a protein (G532R and K595R), the engineered CRISPR/Cas12a_RR protein can recognize unconventional PAM sequences (28, 32). This system was applied for the first time to detect the *katG* G944C (S315T) mutation in isoniazid-resistant *Mtb*. Compared with existing methods such as DNA deep sequencing and TaqMan qPCR, RPA effectively amplified the target gene from 0.1 ng of *Mtb* genomic DNA, facilitating subsequent detection using the CRISPR/Cas12a_RR system. Notably, the CRISPR-based approach demonstrated the ability to detect the *katG* G944C mutant target DNA at concentrations as low as 1%, thereby offering superior sensitivity compared with conventional methods. Owing to the presence of heterogeneous drug resistance, patients with TB may have both drug-sensitive and MDR *Mtb* strains. Low-frequency drug-resistant mutations are often missed by conventional methods, thereby affecting the accuracy of drug-resistance testing. Therefore, to detect drug-resistant *Mtb* in clinical samples using the CRISPR/Cas12a_RR system, it is essential to eliminate interference signals from sensitive *Mtb* strains to enhance the detection efficiency of the method.

Because the CRISPR/Cas12a_RR protein can recognize single-base pair mutations without the restriction of traditional TTTN (N: A, T, G, C) PAM, we designed several single-base pair-specific crRNAs around the *katG* G944C (S315T) mutation site that perfectly matched the mutant sequences. When the target bp is absent, this synthetic mismatch generates bp bubbles, leading to more stringent recognition at the target bp position and allowing specific crRNAs to distinguish single-base pairs in the target DNA sequence (43). We identified a crRNA that can distinguish between the *katG* G944C (S315T) mutation in isoniazid-resistant *Mtb* and the wild-type *katG* within 15 min. With an increase in the detection time and nucleic acid concentration, the interference signal generated by *katG* wild-type *Mtb* increased, which could be modulated by controlling the reaction time and total nucleic acid concentration. A mutation at position 944 of the *katG* gene in isoniazid-resistant *Mtb* resulted in a change in its restriction site at the same position. We cleaved the *katG* wild-type gene using a rapid restriction enzyme specific to the *katG* wild-type gene. This not only increased the ratio of *katG* G944C (S315T) nucleic acids in the DNA template but also greatly increased the nucleic acid content in isothermal amplification products, which was beneficial for subsequent detection.

The primary advantages of the CRISPR/Cas12a_RR detection system are its high sensitivity, specificity, and rapid turnover (43). Compared with PCR, RPA can achieve an amplification time of 20 min for double-stranded DNA while also applying a simplified nucleic acid amplification process. In this study, the target genes were amplified by RPA with 0.1 ng of the *Mtb* genome. Furthermore, the single-nucleotide specificity of the CRISPR/Cas12a_RR detection system was validated for the detection of isoniazid-resistant TB-related mutation sequences. Even when the target gene content was 1% of the total sample, the gene could be effectively recognized. The CRISPR/Cas12a_RR assay showed higher specificity than conventional diagnostic methods such as TaqMan qPCR and DNA sequencing. These findings indicate that the CRISPR/Cas12a_RR assay is efficient and feasible for diagnosing isoniazid-resistant *Mtb*. However, this study has some limitations. First, in this study, isoniazid resistance in *Mtb* is primarily caused by *katG* mutations at G944C (S315T), G944A (S315N), and −15 inhA. Second, we detected only clinical isolates of isoniazid-resistant *Mtb* associated with the highly prevalent *katG* G944C (S315T) mutation. Future research should focus on analyzing larger sample sizes and exploring multiple drug resistance sites.

In conclusion, this study presents the first CRISPR/Cas12a_RR system CRISPR/Cas12a_RR-based detection system with high specificity and sensitivity in diagnosing isoniazid-resistant *Mtb*. Even when the target gene constitutes merely 1% of the total sample, it can still be effectively identified and validate its detection performance using clinical isolates. Compared with TaqMan qPCR and DNA sequencing methods, the CRISPR/Cas12a_RR system offers a simplified detection process, requiring only blue fluorescent light with an emission wavelength of 485 nm and a CRISPR lateral flow test strip to detect drug-resistant *Mtb*. These features make it particularly suitable for point-of-care testing at remote hospitals and testing facilities with limited resources and large patient volumes. The entire detection process can be completed within 60 min. Furthermore, the high sensitivity and specificity of the CRISPR/Cas12a_RR system demonstrate significant potential for the timely diagnosis and treatment of patients with isoniazid-resistant *Mtb*. Future studies should assess the performance of the proposed system across a broader range of isoniazid-resistant *Mtb* strains, including those carrying less prevalent gene mutations.

## Data Availability

All data generated or analyzed during this study are included in this published article and its supplementary information files.

## SUPPLEMENTAL MATERIAL

Supplemental Material spectrum.00165-25-s0001.docx Figure S1; Tables S1 to S3.

ASM does not own the copyrights to Supplemental Material that may be linked to, or accessed through, an article. The authors have granted ASM a non-exclusive, world-wide license to publish the Supplemental Material files. Please contact the corresponding author directly for reuse.
