# Supplementary material for: Application of engineered CRISPR/Cas12a variants with altered protospacer adjacent motif specificities for the detection of isoniazid resistance mutations in Mycobacterium tuberculosis
Source: Microbiol Spectr. 2025 Sep 3;13(10):e00165-25. doi: 10.1128/spectrum.00165-25 (PMC12502725; doi:10.1128/spectrum.00165-25)
Supplement: Supplemental Material — Figure S1; Tables S1 to S3. [file spectrum.00165-25-s0001.docx]

**Supplementary Figures**





**Supplement Figure 1**. **The AciI endonuclease can cleave the wild gene into multiple small fragments**. The DNA template was digested with AciI endonuclease, and the digested template was examined by electrophoresis on a 2% agarose gel.

**Supplementary Tables**

**Supplementary Table 1. Core sequences of synthetic mismatched crRNA.**

| crRNA name | crRNA sequence |
| --- | --- |
| *KatG* WT-crRNA1 | gucgggguguucguccauacgac |
| *KatG* **G**944**C**-crRNA1 | caccggcaucgaggucguaugga |
| *KatG* **G**944**C**-crRNA2 | gguggugaucgcguccuuaccgg |
| *KatG* **G**944**C**-crRNA3 | augccgguggugaucgcguccuu |
| *KatG* **G**944**C**-crRNA4 | gaugccgguggugaucgcguccu |
| *KatG* **G**944**C**-crRNA5 | uacgaccucgaugccggugguga |
| *KatG* **G**944**C**-crRNA6 | uccauacgaccucgaugccggug |

crRNA: CRISPR guided-RNA. The PAM sequences corresponding to crRNA1-6 are TCAC, TGCC, CTCG, CCTC, TCCA and TTCG, respectively.

**Supplementary Table 2. Primers used in this study.**

| Primer name | Primer sequence |
| --- | --- |
| *KatG* F | tcacactttcggtaagacccatg |
| *KatG* R | aaggtatctcgcaacgggac |
| *KatG* RPA-F1 | cgctggagcagatgggcttgggctggaagagc |
| *KatG* RPA-F2 | ggcttgggctggaagagctcgtatggcaccgg |
| *KatG* RPA-F3 | agctcgtatggcaccggaaccggtaaggacgcg |
| *KatG* RPA-F4 | cgtatggcaccggaaccggtaaggacgcgatcacc |
| *KatG* RPA-R1 | gtattgccaagcgccagcagggctcttcgtcagc |
| *KatG* RPA-R2 | ctcttcgtcagctcccactcgtagccgtacag |
| *KatG* RPA-R3 | ctcccactcgtagccgtacaggatctcgagg |
| *KatG* RPA-R4 | cactcgtagccgtacaggatctcgaggaaactg |

RPA: recombinase polymerase amplification.

**Supplementary Table 3. TaqMan qPCR amplified primer and TaqMan probe sequences**

| Primer name | Primer sequence |
| --- | --- |
| *KatG* TaqMan qPCR F | agcagatgggcttgggctg |
| *KatG* TaqMan qPCR R | cccatttcgtcggggtgtt |
| *KatG* TaqMan Probe1 | taaggacgcgatcaccaccggca |
